# EZH2 Phosphorylation Promotes Self-Renewal of Glioma Stem-Like Cells Through NF-κB Methylation

**DOI:** 10.3389/fonc.2019.00641

**Published:** 2019-07-16

**Authors:** Hailong Liu, Youliang Sun, Xueling Qi, Renata E. Gordon, Jenny A. O'Brien, Hongyu Yuan, Junping Zhang, Zeyuan Wang, Mingshan Zhang, Yongmei Song, Chunjiang Yu, Chunyu Gu

**Affiliations:** ^1^Department of Neurosurgery, Sanbo Brain Hospital Capital Medical University, Beijing, China; ^2^Department of Neurosurgery, Chinese People's Liberation Army General Hospital, Beijing, China; ^3^School of Basic Medical Science, Capital Medical University, Beijing, China; ^4^Department of Neuropathology, Sanbo Brain Hospital Capital Medical University, Beijing, China; ^5^Cancer Biology Program, Fox Chase Cancer Center, Philadelphia, PA, United States; ^6^Department of Internal Medicine, Temple University Health System, Philadelphia, PA, United States; ^7^State Key Laboratory of Molecular Oncology, National Cancer Center/Cancer Hospital, Chinese Academy of Medical Sciences and Peking Union Medical College, Beijing, China; ^8^School of Pharmacy, Temple University, Philadelphia, PA, United States

**Keywords:** cancer stem-like cells, glioma, EZH2 phosphorylation, NF-κB methylation, self-renewal

## Abstract

Cancer stem-like cells (CSCs) is a cell population in glioma with capacity of self-renewal and is critical in glioma tumorigenesis. Parallels between CSCs and normal stem cells suggest that CSCs give rise to tumors. Oncogenic roles of maternal embryonic leucine-zipper kinase (MELK) and enhancer of zeste homolog 2 (EZH2) have been reported to play a crucial role in glioma tumorigenesis. Herein, we focus on mechanistic contributions of downstream molecules to maintaining stemness of glioma stem-like cells (GSCs). Transcriptional factor, NF-κB, co-locates with MELK/EZH2 complex. Clinically, we observe that the proportion of MELK/EZH2/NF-κB complex is elevated in high-grade gliomas, which is associated with poor prognosis in patients and correlates negatively with survival. We describe the interaction between these three proteins. Specifically, MELK induces EZH2 phosphorylation, which subsequently binds to and methylates NF-κB, leading to tumor proliferation and persistence of stemness. Furthermore, the interaction between MELK/EZH2 complex and NF-κB preferentially occurs in GSCs compared with non-stem-like tumor cells. Conversely, loss of this signaling dramatically suppresses the self-renewal capability of GSCs. In conclusion, our findings suggest that the GSCs depend on EZH2 phosphorylation to maintain the immature status and promote self-proliferation through NF-κB methylation, and represent a novel therapeutic target in this difficult to treat malignancy.

## Introduction

Glioma is the most common primary malignant brain tumor, accounting for almost 40% of primary central nervous system tumors, of which glioblastoma (GBM) is the leading cause of mortality (1, 2). The current therapeutic strategy for glioma is typically surgical resection followed by chemotherapy and radiation. Unfortunately, in spite of significant advances in diagnostic and therapeutic approaches, the median survival of GBM patients remains low and about 14.2 months (3, 4). This could be attributed to traditional treatment producing limited efficacy on the subset of tumor cells with the potential to self-renew, termed cancer stem-like cells (CSCs) (5, 6). Recent studies indicated the similarity between normal stem cells and CSCs in self-renewal regulation (7–9). The pathways mediating the stemness of normal neural stem cells (NSCs), such as Notch, WNT/β-catenin and Hedgehog signaling, are also critical in glioma stem-like cells (GSCs) to drive tumorigenicity. Recently, establishment of novel therapeutic approaches with the goal of not only reducing the tumor burden, but also targeting GSCs evolved (10–13). However, molecular mechanisms driving long-term self-proliferation of GSCs are not completely understood. Comprehensive understanding of biological behaviors of GSCs is required for development of targeted therapy, which spares non-malignant NSCs intact.

Maternal embryonic leucine zipper kinase (MELK), a member of the snf1/AMPK family, has been reported to be enriched in stem cells at embryonic stage with functions of embryogenesis and cell cycle modulation (14–16). Recent studies have identified the expression of MELK in a large scale of cancers (17–19). Several reports indicate that MELK is involved in stemness maintenance and cell cycle regulation in tumorigenesis. Intriguingly, the survival of NSCs isn't affected by MELK knockdown, suggesting that MELK activation is specific to tumor (20). Based on our previous work, MELK contributed to the induction of stem-like features in GBM via the MELK/c-JUN or MELK/FOXM1 pathway. This facilitated tumor relapse and chemoradioresistance (21, 22). Though several studies highlighted MELK function in malignancy programming, the mechanism associated with its epigenetic mediation remains unclear.

Furthermore, enhancer of zeste homolog 2 (EZH2), a protein member of the c-Myc/HBXIP/Hotair/LSD1 complex, also plays a vital role in the differentiation of stem cells during development via epigenetic modification (23–26). According to the existing data, elevated expression of EZH2 has been detected in a wide spectrum of cancers, especially in brain tumors (27–31), suggesting its tumorigenic potential (32). It has been observed that EZH2 functions in epigenetic modulation through methylated histones (33, 34). Additionally, increased evidence indicates that downstream transcription molecules in EZH2 pathway facilitate AKT mediated phosphorylation (35, 36). Consistent with this, downstream factors including STAT3 and GATA4 have been co-immunoprecipitated by EZH2 (37–39). Nevertheless, NF-κB activity in EZH2 phosphorylation and its potential role in GBM tumorigenicity remain unknown.

Previously, we showed that MELK and EZH2 were required for recurrent GBM to retain its proliferative and invasive capacity (21, 22). It is not known whether EZH2 phosphorylation can be mediated by upstream MELK and whether the process underlying phosphorylation is indispensable for tumor progression. In the current study, we demonstrate that MELK/EZH2/NF-κB signaling, activated in human high-grade gliomas, represents a key functional hallmark of GSCs and results in the poor prognosis of GBM patients. MELK-mediated EZH2 phosphorylation induces the methylation of NF-κB in GSCs, thereby facilitating the transcription activity. Finally, ablation of MELK/EZH2/NF-κB axis can impair stemness of GSCs and promote differentiation, leading to a reduced tumor burden. This work identifies a potential therapeutic target for GSCs in GBM.

## Materials and Methods

### Human Glioma Samples

The glioma specimens were obtained from 375 patients undergoing craniotomy in Sanbo Brain Hospital Capital Medical University from January 2011 to September 2016. None of patients received radiation or chemotherapy prior to craniotomy. Tissues were harvested during surgery, and subsequently frozen and stored in liquid nitrogen. All cases were diagnosed by two independent neuropathologists. Clinicopathological and neuroimaging parameters were shown in Table S1. Survival analysis was performed based on the follow-up information. This study was carried out in accordance to the ethics committee protocols of Sanbo Brain Hospital Capital Medical University. All patients gave written informed consent in accordance to the Declaration of Helsinki.

### Cell Culture

Primary GSCs were isolated from human GBM samples shortly after operations. GBM tissues were chopped and digested in a solution containing papain (20 U/ml) and DNase I (4%) into a single cell suspension. CD133^+^/CD44^+^ (Abcam biotechnology) expressing cells were purified by FACS and cultured at 1 × 10^7^ /ml in DMEM/F12 medium supplemented with B27 (2%), bFGF (20 ng/mL), EGF (50 ng/mL), L-glutamine (1%), and penicillin/streptomycin (1%). Control cells were cultured in 10% FBS-containing DMEM media. To sort the NSCs, hippocampal tissues were digested and dissociated into single cell suspension for further FACS sorting upon CD133 expression. The obtained cells were cultured in Neurobasal medium containing B27 (2%), bFGF (20 ng/mL), EGF (50 ng/mL), L-glutamine (1%), and penicillin/streptomycin (1%). The cortex of fetal or adult mice were digested with 0.25% trypsin and resuspended at 1 × 10^7^ /ml in Neurobasal medium supplemented with B27 (2%), L-glutamine (1%), and penicillin/streptomycin (1%) to isolate the neurons (binding part) and gliocytes (suspended part). The suspended gliocytes were separated in DMEM supplemented with 10% FBS.

### Immunohistochemistry and Immunofluorescence

All sections were prepared from paraffin-embedded tissues and sliced into 5 μm sections. Specimen sections were stained with the anti-MELK (CST2274), EZH2 (CST5246), NF-κB (CST8242), or P65 (SAB4502610) antibodies (1: 100) at 4°C overnight, and then washed and incubated with secondary antibodies for 1 h. The number of positive stained cells was blindly determined by two pathologists based on counting of 500 nuclei in 4 high-magnifying (400×) representative fields and reported as percentage. Histological subtypes were identified by two independent pathologists. Frozen sections, neurospheres, or dissociated GSCs were blocked for 1 h in normal goat serum and subsequently incubated with anti-MELK, EZH2, NF-κB, CD133, Nestin, GFAP, or Ki-67 antibodies (1:200) overnight at 4°C and stained with FITC secondary antibody at room temperature for 1 h. All samples were counterstained with DAPI.

### Western Blot and Co-immunoprecipitation Analysis

Fresh tissues or cells and were lysed in splitting buffer (Supplementary Materials for details) supplemented with protease inhibitor, incubated on ice for 15 min and then centrifugated at 3,000 g at 4°C for 10 min. Whole protein (50 μg) was separated by 10% SDS-PAGE gel and then transferred to PVDF membranes. The membranes were blocked in 5% BSA solution and subsequently incubated with associated antibodies overnight at 4°C, and then probed with secondary antibodies for 1 h at room temperature. Anti-MELK, EZH2, and NF-κB antibodies or anti-SUV391H1 and p-KMT6/EZH2 antibodies cross-linked with protein A/G beads were incubated with 500 μg of lysates from cells overnight at 4°C, respectively. The IgG was used as the negative control. Then protein was eluted from beads and added into the 10% SDS-PAGE gel for electrophoresis. Immunoblotting for the indicated antibodies was performed.

### qPCR

RNA was extracted from tumor cells or GSCs using Trizol reagent according to the manufacture's procedures (Invitrogen). For qPCR, cDNA was synthesized by using iScript reverse transcriptase from 2 μg of total RNA. The relative quantification of mRNA was determined by using SYBR-green on the BIORAD qPCR system and normalized for the expression of GAPDH mRNA.

### Colony Formation Assay

Dissociated GSCs infected with shMELK, shEZH2, shNF-κB, and scrambled shRNA were seeded in 60 mm dishes coated with PDL and cultured in DMEM/F12 without FBS. Colonies were prepared using the same method and then cultured in the presence of DZNep, ACHP, and DMSO. After 10 days, the colonies were fixed with 4% paraformaldehyde and immersed into crystal violet.

### Transplantation and Drug Administration

All animal experiments were performed in accordance to the national guidelines at Capital Medical University and approved by our institutional ethics committee. Dissociated GSCs infected with shMELK, shEZH2, shNF-κB, and scrambled shRNA were subcutaneously injected into the left (experimental) and right (control) flank of 6–8-week-old male SCID mice. Tumor volumes were measured every 3 days and calculated according to the following formula, V (mm^3^) = 1/2 (L × W^2^). Tumor sections were stained against Ki-67, Nestin, GFAP, and P65 antigens. For the drug administration, engraftments were checked on the 7th day after transplantation and mice with equivalent volumes were randomized into several groups (*n* = 6 per group) for gavage treatment: OTSSP167, DZNep, ACHP, or MCT. Measurement of tumor growth was conducted, and immunostaining for Ki-67 was tested.

### Statistical Analysis

Data statistical handling was performed using SPSS 19.0 and Graphpad Prism 7.0 software, and values were shown as *mean* ± *SD* with error bars representing SEM. An unpaired *t*-test was utilized between two groups, and comparison of mean values between multiple groups was evaluated by one-way ANOVA. Associations of staining indexes were analyzed by Pearson test. To analyze the survival data, overall or progressive-free survival (PFS) curve was adopted using Kaplan-Meier estimate, and variate prognostic factors were estimated by Cox proportional model. For all statistical methods, *p* < 0.05 was considered significant.

## Results

### MELK/EZH2/NF-κB Is Highly Expressed in Human GBM Associated With Poor Survival

**Figure 1:**
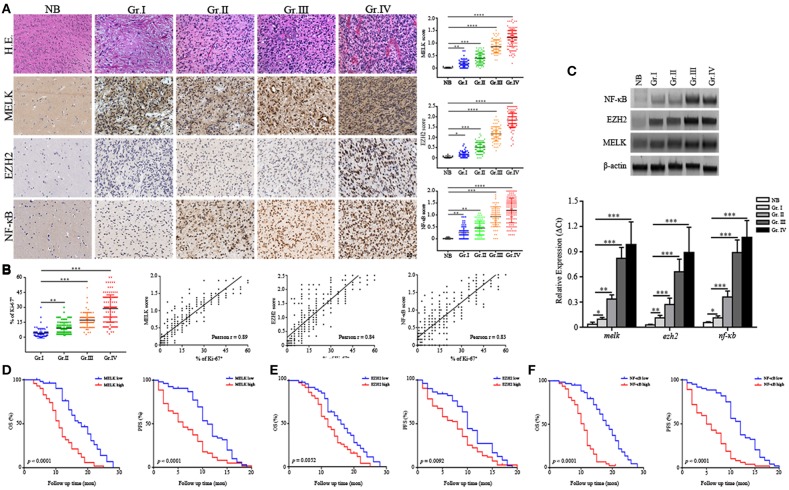
MELK, EZH2, and NF-κB are highly expressed in high-grade glioma. **(A)** Representative IHC panels across four grades of glioma and adjacent normal tissues (200×) showing the expression of MELK, EZH2, and NF-κB. **(B)** Pearson analysis showing the association of strong MELK/EZH2/NF-κB expression with high Ki-67 index (Pearson *r* = 0.89, 0.84, and 0.83, respectively). **(C)** Up, immunoblotting analysis showing protein levels of MELK, EZH2, and NF-κB. Down, qPCR analysis displaying the mRNA level of *melk, ezh2*, and *nf*-κ*b* in surgical glioma samples. The experiments were repeated three times. **(D–F)** The postsurgical OS and PFS curves evaluated by Kaplan-Meier methods among GBM patients showing poor survival was associated with high expression of MELK, EZH2, or NF-κB (low, the IHC score of MELK, EZH2, or NF-κB was <1.2, 1.5, and 1.2, respectively; high, the IHC score was more than 1.3, 1.6, and 1.3, respectively). *p < 0.05, **p < 0.01, ***p < 0.001, ****p < 0.0001.

Human glioma samples in this cohort comprised four grades including WHO Grade I (*n* = 65), Grade II (– = 108), Grade III (*n* = 77), and Grade IV (*n* = 125) and adjacent normal brain tissues (*n* = 9). We examined MELK, EZH2, and NF-κB expression by immunohistochemistry and demonstrated that MELK, EZH2, and NF-κB were abundantly enriched in the nuclei of high-grade gliomas with the average score ranking from 1.2 to 1.8, compared with the low-grade and normal adjacent tissues (Figure 1A). The expression correlation analysis for each these markers was positive based on the Pearson estimates (Figure S1A). As presented in Figure 1B, association was demonstrated between increased expression of MELK, EZH2, NF-κB, and Ki-67 index. This association was statistically significant supporting a critical role of these proteins in proliferation of tumor cells (Pearson *r* = 0.89, 0.84, and 0.83, respectively). To further confirm the expression profiles, we examined expression of MELK, EZH2, and NF-κB at protein and RNA levels and found that the EZH2 and NF-κB expression was markedly increased in the surgical GBM samples utilizing the immunoblotting and qPCR investigation though there was not detectable difference at MELK protein level (*p* < 0.01 and *p* < 0.001, Figure 1C).

Further, survival analysis was performed to analyze association of MELK/EZH2/NF-κB with prognosis of glioma patients. Among the 375 cases, the median follow-up time was 53.5 months (range, 1.0 ~ 68.5 months) with 193 (51.46%) surviving patients during this period. Patients with lower histologic expression of MELK/EZH2/NF-KB were less likely to succumb to glioma compared with medium or high expression (Table S1). Overall survival (OS) and PFS Kaplan-Meier curves were generated to compare survival rates based on histologic expression of MELK, EZH2, or NF-κB. These curves demonstrated that GBM patients with stronger expression level of MELK, EZH2, or NF-κB suffered from reduced postoperative OS and PFS (Figures 1D–F). Conversely, patients with low levels of MELK/EZH2/NF-κB and low-grade gliomas had increased survival (Figures S1B,C). In addition, both the odd ratio (OR) data from Logistic analysis and Cox proportional model indicated the MELK/EZH2/NF-κB expression as significant prognostic indicator (Tables S2, S3). Next, we investigated the relationship between clinicopathological parameters and the expression to evaluate the prognostic factors, which demonstrated that remarkable enrichment of MELK, EZH2, and NF-κB was associated with higher grade gliomas, basal ganglia/brainstem, and heterogeneous MRI enhancement (Table S2). Thus, this data indicates that MELK, EZH2, and NF-κB are associated with decreased postsurgical survival of GBM patients, suggesting therapeutic potential of targeting these molecules.

### MELK, EZH2, and NF-κB Are Enriched in GSCs

**Figure 2:**
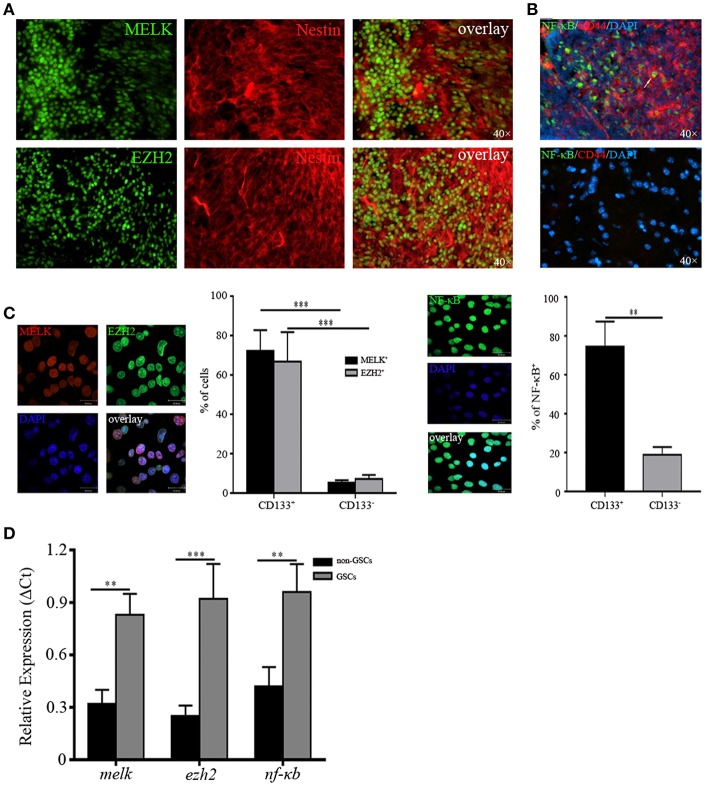
MELK, EZH2, and NF-κB are enriched in GSCs. **(A)** Frozen sections from human GBM samples immunostained for MELK, EZH2, and Nestin (400×) displaying the co-location of MELK/EZH2 and Nestin. **(B)** Immunofluorescence staining against NF-κB and CD44 in human GBM (400×) showing the co-location of CD44 and NF-κB. The white arrow represented one cell which was double positive for NF-κB and CD44. **(C)** Cells sorted from the fresh human GBM by FACS utilizing CD133/CD44 surface markers were immunostained for MELK, EZH2, and NF-κB. **(D)** qPCR analysis showing the high mRNA levels of *melk, ezh2, and nf*-κ*b* in GSCs. The experiments were repeated three times. **p < 0.01, ***p < 0.001.

Having identified upregulated expression of MELK/EZH2/NF-κB in human GBM, we then investigated the location of cells expressing all of above molecules to test their possible function in tumorigenic proliferation and progression. We found that a majority of MELK^+^ or EZH2^+^ cells were co-localized with the putative GSCs marker, Nestin (Figure 2A). Similarly, the extensive overlay of NF-κB^+^ and GSCs marker CD44^+^ was observed throughout GBM tissue (Figure 2B). To check the expression of MELK/EZH2/NF-κB in single cells, we purified the GSCs from surgical specimens by harvesting CD133^+^/CD44^+^ cells via FACS (Figure S2). When cultured in stem cell media, spheres were observed demonstrating the stem-like properties of sphere formation. Sorted cells expressed MELK, EZH2, and NF-κB in nuclei, however little signal was detected in the CD133^−^ group representing non-GSC tumor cells (*p* < 0.01 and *p* < 0.001, Figure 2C). To corroborate the staining results regarding cancer stem-like features in GSCs, we found robust expression of MELK, EZH2, and NF-κB in GSCs sorted from GBM cell-line, U87 and primary human GBM tissues, compared with control cultures (Figure S3). Similarly, dramatically elevated levels of relative *melk, ezh2*, and *nf*-κ*b* mRNA were observed in GSCs, but no significant differences were found in non-GSCs (*p* < 0.01 and *p* < 0.001, Figure 2D). Therefore, for the following experiments, GSCs with CD133/CD44 surface markers were collected by FACS and cultured in the stem cell media. The percentage of MELK, EZH2, and NF-κB in GSCs suggests that they may play a key role in maintenance of stemness.

### EZH2 Is Phosphorylated by MELK in GSCs

**Figure 3:**
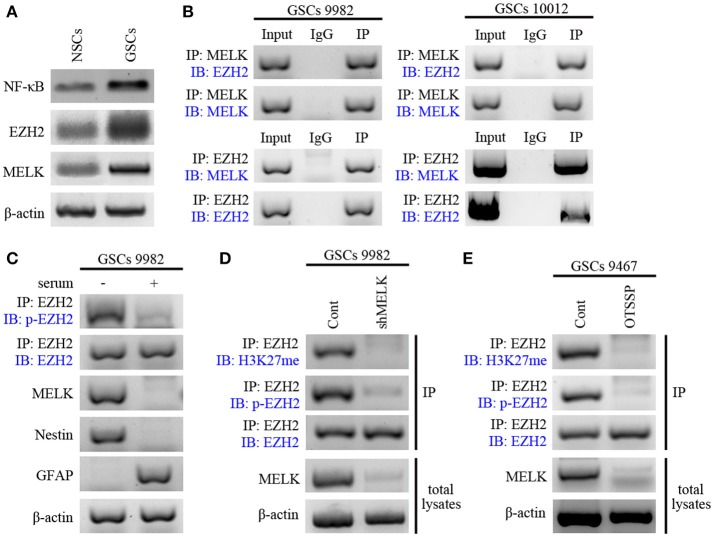
EZH2 is phosphorylated by MELK in GSCs. **(A)** Immunoblotting analysis showing higher expression of MELK, EZH2, and NF-κB proteins in GSCs compared to that in NSCs. **(B)** Immunoblotting analysis showing endogenous MELK bound to EZH2 in GSCs (9,982 and 10,012) following co-IP with MELK antibody. Western blot showing the binding of endogenous EZH2 to MELK in GSCs following co-IP with EZH2 antibody. **(C)** Western blot showing phosphorylation of EZH2 after IP of the lysates with EZH2 antibody in GSCs (9,982) cultured with or without serum. Actin was used as the loading control. **(D)** IP and western blot showing the impaired p-EZH2 activity based on the low expression of H3K27me in GSCs (9,982) infected with shMELK. **(E)** IP and western blot showing impaired p-EZH2 activity based on the low expression of H3K27me in GSCs (9,467) after treatment of OTSSP167. The experiments were repeated three times.

We examined expression of MELK, EZH2, and NF-κB in NSCs during hippocampal development and found the elevated expression (Figure S4). Next, we examined their expression in GSCs compared with NSCs via western blot and found strong enrichment of the three proteins, suggesting the tumor-specific overexpression (Figure 3A). Because of the kinase activity of MELK and the methytransferase activity of EZH2, we examined whether endogenously expressed MELK could physically bind to EZH2 by coimmunoprecipitation (co-IP, Figure 3B). EZH2 and MELK co-localized in GSCs and could be co-precipitated from GSCs lysate using either MELK antibody (Figure 3B, left) or EZH2 antibody (Figure 3B, right). This led us to investigate whether stem-like features of GSCs relied on the MELK/EZH2 complex. Moreover, we asked whether MELK could phosphorylate the downstream EZH2 due to its kinase activity. We immunoblotted the pattern of phosphorylated EZH2 (p-EZH2) in the whole compound precipitated by MELK antibody demonstrating that EZH2 phosphorylation required the MELK activity in GSCs (Figure 3C).

EZH2 has been previously reported as the catalytic subunit of PRC2 and to trimethylate the Lys^27^ of H3 histone (H3K37me3) during tumorigenesis (35, 40). To verify the role of phosphorylated EZH2 in glioma progression, we examined H3K27-methylation in GSCs in the presence or absence of lentivirus carrying shRNA against MELK or pharmacological inhibitors. As expected, MELK knockdown dramatically suppressed the p-EZH2 expression and consecutively resulted in the reduction of H3K27me level to about 20% of the non-targeted group (Figure 3D) suggesting that EZH2 expression and its phosphorylation activity require the presence of MELK. These results were also achieved with treatment of GBM cells with MELK inhibitor, OTSSP167, which significantly diminished MELK substrates, debrin-like (DBNL), and proteasome alpha subunit 1 (PSMA1) (41). As shown in Figure 3E, both p-EZH2 and H3K27me profiles were significantly decreased following treatment with OTSSP167. Observed results are consistent with previous studies demonstrating phosphorylation of EZH2 was essential to activate downstream signaling, suggesting the requirement of MELK to modify EZH2 in GSCs. Thus, these data suggest that interaction of MELK is required for EZH2 phosphorylation, a known player in tumorigenicity.

### MELK/EZH2 Complex Causes NF-κB Methylation

**Figure 4:**
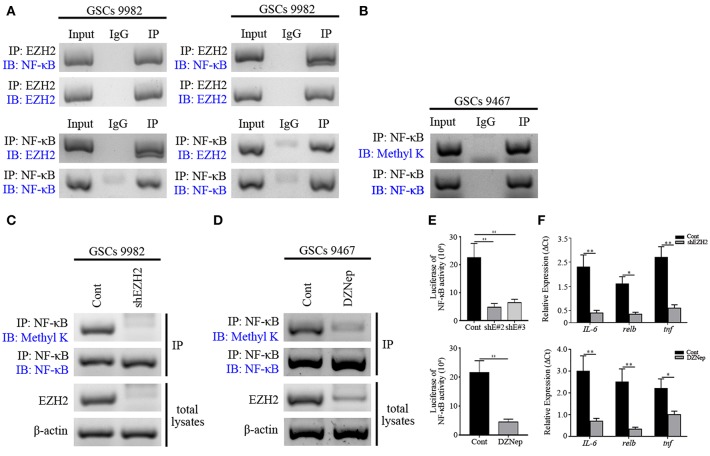
NF-κB is methylated by EZH2 in GSCs. **(A)** Western blot analysis showing binding of endogenous EZH2 to NF-κB in GSCs (9,982) following co-IP with EZH2 antibody. Western blot analysis showing binding of endogenous NF-κB to EZH2 after co-IP with NF-κB antibody. **(B)** Immunoblotting analysis showing the expression of Methyl K after IP of the lysates with NF-κB antibody. **(C)** Immunoblotting assay showing the expression of the Methyl K following IP of the lysates from GSCs (9,982) receiving the shEZH2 or scrambled shRNA infection with NF-κB antibody. **(D)** Immunoblotting assay showing the expression of Methyl K following IP of the lysates from GSCs (9,467) with NF-κB antibody after GSCs receiving the treatment of DZNep. **(E)** Responsive luciferase reporter assay showing the inhibited methylation activity of NF-κB in shEZH2 expressing and DZNep treatment group. **(F)** qPCR analysis determining the decreased mRNA levels of *IL-6, relb*, and *tnf* in EZH2 deficient GSCs. The experiments were repeated three times. *p < 0.05, **p < 0.01. shE#2, shEZH2#2; shE#3, shEZH2#3.

Having observed a functional interaction of MELK and EZH2 in GSCs as well as previously recognized methyltransferase capability of EZH2 (35), we further examined whether EZH2 methylated the downstream transcription factor, NF-κB. First, co-IP of EZH2 and NF-κB was performed on GSCs lysates. This showed that robust binding occurred between the two proteins in GSCs (Figure 4A). In contrast, addition of serum into the GSCs culture system decreased the binding of EZH2 to NF-κB, indicating that EZH2/NF-κB interaction was associated with stem-like preference, because the factors in serum stimulated differentiation of GSCs. We found the strong expression of pan-methyl lysine antibody (Methyl K) in GSCs following immunoprecipitation with NF-κB antibody, which suggested that EZH2 played a critical role in NF-κB methylation (Figure 4B). To confirm the role of EZH2 in NF-κB methylation, EZH2/NF-κB complex was pulled down with NF-κB antibody and immunoblotted with Methyl K. GSCs following shEZH2 carrying lentivirus infection or treatment of DZNep, presented significantly lower level of Methyl K compared to uninfected/untreated controls (Figures 4C,D). However, to validate the condition of NF-κB, we performed western blot to check its expression and no change in NF-κB expression was observed in EZH2-deficient GSCs (Figure S5). Furthermore, based on the vital transcriptional function of NF-κB, we next investigated the NF-κB activity by NF-κB responsive luciferase assay and qPCR analysis. A dominant downregulation of responsive luciferase was detected in both shEZH2-infected and DZNep-treated GSCs (*p* < 0.01), which indicated that NF-κB activity was damaged in EZH2 deficiency status (Figure 4E). Similarly, the prominent reduced mRNA expression of NF-κB targeted genes (*IL-6, relb*, and *tnf*) was observed in the EZH2 deficient group (*p* < 0.05 and *p* < 0.01), suggesting that insufficient EZH2 led to the dysfunction of NF-κB activity (Figure 4F). Taken together, the above data demonstrates that NF-κB methylation is induced by phosphorylated EZH2 facilitating the transcription activity in GSCs.

### NF-κB Targeting Impairs Glioma Stemness

**Figure 5:**
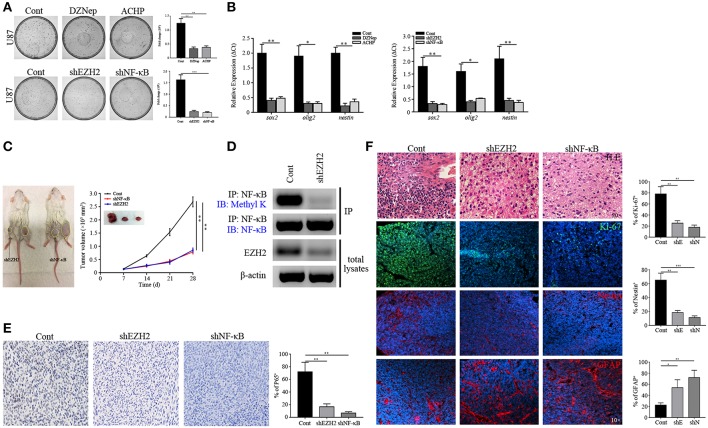
Stemness maintenance is required for NF-κB activity. **(A)** Colony formation assay showing the decreased proliferation of GSCs sorted from U87 cells after EZH2/NF-κB inhibition. **(B)** qPCR analysis determining the mRNA expression of *sox2, olig2*, and *nestin*. **(C)** The growth curves showing the growth rate of xenografts derived from the primary GSCs expressing shEZH2 and shNF-κB. **(D)** Immunoblotting assay showing the expression of Methyl K following IP of GSCs lysates with NF-κB antibody in the shEZH2 expressing GSCs derived tumors. **(E)** IHC staining showing the expression of P65 in shEZH2 and shNF-κB infected GSCs derived tumors (200×). **(F)** H.E. staining showing the Grade 2 morphology in the EZH2 and NF-κB knockdown group. Immunostaining images showing the expression of Ki-67, Nestin and GFAP in subcutaneous xenograft samples derived from scrambled shRNA, shEZH2, and shNF-κB GSCs. The experiments were repeated three times. *p < 0.05, **p < 0.01, ***p < 0.001. shE, shEZH2; shN, shNF-κB.

To test the contribution of the MELK/EZH2/NF-κB axis on self-proliferation of GSCs, we performed colony formation and found only about 30% of the control after treatment of DZNep and ACHP (*p* < 0.01, Figure 5A). EZH2 or NF-κB depletion significantly attenuated the proliferation of GSCs, reflected by the decreased ratio of colony formation (*p* < 0.001, Figure 5A). Because of the location of MELK, EZH2, and NF-κB in GSCs, we then assessed the stemness gene expression by qPCR in GSCs, in which the EZH2/NF-κB expression was abolished. As shown in Figure 5B, genes associated with stemness of GSCs were significantly downregulated in the EZH2/NF-κB deficient group (*p* < 0.05 and *p* < 0.01).

To test whether our findings were consistent with *in vitro* setting, we next generated the GSCs-bearing mice to evaluate effects of EZH2 and NF-κB suppression by utilizing appropriate inhibitors. It revealed that DZNep and ACHP reduced tumor growth in mice (*p* < 0.01 and *p* < 0.001, Figure S6A), confirming our *in vitro* findings. Similarly, EZH2 and NF-κB silencing attenuated the GBM proliferation, compared to the control group (*p* < 0.05, Figure 5C). These data demonstrated that ablation of EZH2/NF-κB dramatically suppressed GBM progression. Having collected the tumor tissues, western blot and IP were performed to detect the expression of Methyl K, which suggested that deletion of EZH2 inhibited the NF-κB methylation (Figure 5D). Given a role of P65, the subunit of NF-κB, as a bioactivation marker in NF-κB pathway (42), we assessed whether P65 expression was declined in human-derived xenografts from the shEZH2-expressing GSCs (Figure 5E). Next, frozen tumor tissues were prepared to examine the proliferation and stemness by immunostaining. The incompact density of tumor cells, lack of pathological vessels and necrosis indicated the low-grade features in shEZH2 and shNF-κB infected GSCs-driven tumors (Figure 5F). Only a fraction of EZH2 and NF-κB deficient tumors were positive for Ki-67 (*p* < 0.01, Figure 5F). Consistent with previously shown data, the EZH2 and NF-κB deficient samples displayed no obvious expression of Nestin, but an extensive level of differentiation evidenced by high number of GFAP-expressing cells (*p* < 0.01 and *p* < 0.001, Figure 5F). Similar results assessing proliferation were obtained in the DZNep and ACHP treatment group (*p* < 0.01 and *p* < 0.001, Figure S6B). Therefore, these data support the hypothesis that EZH2 promotes the tumorigenesis stemness through NF-κB methylation.

### MELK Is Essential for EZH2/NF-κB Complex Activity

**Figure 6:**
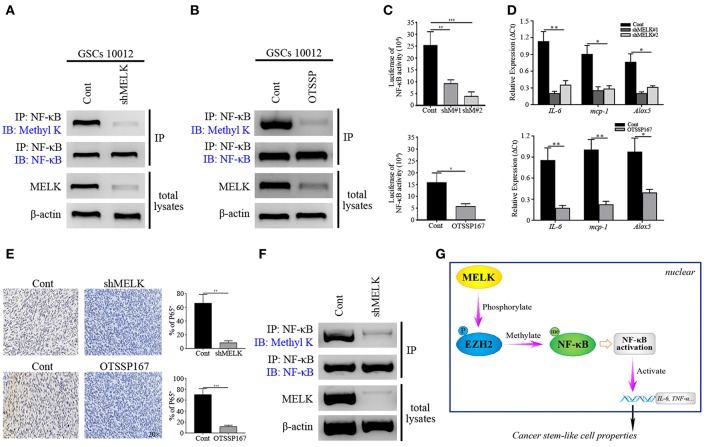
MELK deficiency suppresses the function of NF-κB. **(A)** Immunoblotting analysis showing the expression of Methyl K following IP of GSCs lysates with NF-κB antibody after GSCs (10,012) receiving shMELK infection. **(B)** Immunoblotting analysis presenting the expression of Methyl K following IP of GSCs lysates with NF-κB antibody after GSCs (10,012) receiving the treatment of OTSSP167. **(C)** Responsive luciferase reporter assay showing the inhibited NF-κB activity in MELK deficiency condition. **(D)** CHIP-qPCR analysis showing the mRNA levels of *IL-6, mcp-1*, and *Alox5* after MELK inhibition. **(E)** IHC staining showing the expression of P65 in shMELK infected and OTSSP167 treated GSCs derived tumors (200×). **(F)** Immunoblotting assay showing the suppressed expression of Methyl K following IP of xenografts lysates with NF-κB antibody in shMELK expressing GSCs derived tumors. The experiments were repeated three times. **(G)** The schematic model illustrates the mechanism that MELK-mediated EZH2 phosphorylation methylates NF-κB in GSCs (P, phosphorylation; Me, methylation). *p < 0.05, **p < 0.01, ***p < 0.001. Abbreviations: shM#1, shMELK#1; shM#2, shMELK#2.

Since MELK plays an important role in EZH2 activation in GSCs, we next investigated whether MELK could facilitate the EZH2/NF-κB interaction. Protein complex was pulled down by NF-κB antibody and then immunoblotted against Methyl K, showing that methylated NF-κB status relied on the MELK activity (Figure 6A). Similar phenomenon was confirmed by IP of lysates from GSCs treated with OTSSP167 (Figure 6B). To evaluate the NF-κB activity after blocking MELK, we performed the luciferase report assay and found an obvious reduction of luciferase response in experimental group (*p* < 0.01 and *p* < 0.001, Figure 6C). As a transcriptional factor, NF-κB targeted gene expression including *IL-6, mcp-1*, and *Alox5* was analyzed by CHIP-qPCR. As shown in Figure 6D, the downstream gene expression was dramatically decreased in experimental group (*p* < 0.05 and *p* < 0.01), supporting that the NF-κB transcriptional feature was compromised by MELK inactivation. Additionally, to explore the effects of MELK inhibition on the proliferation of GSCs, spheres assay was performed by immunostaining of Ki-67/Nestin to demonstrate that MELK inhibition resulted in compromised proliferation (*p* < 0.05 and *p* < 0.01, Figure S7A).

We next generated cohorts of mice bearing tumors derived from shMELK expressing GSCs to further evaluate the effects of MELK/EZH2/NF-κB signaling on GBM growth. The tumor burden decreased dramatically following MELK knockdown, and even treatment with IL-1 couldn't rescue the growth (*p* < 0.01, Figure S7B). The xenograft samples were collected and analyzed by H&E. staining to show the decreased proliferation region without necrosis and vessels (Figure S7C). Additionally, the GBM stemness was impaired, and complemented with enhanced differentiation, evidenced by the low-level of Nestin and high number of GFAP-expressing cells across the tumor (*p* < 0.05 and *p* < 0.01, Figure S7C). Decreased tumor volume and Ki-67 index were achieved after the OTSSP167 and ACHP treatment (*p* < 0.001, Figure S8), which suggested that MELK/EZH2/NF-κB axis was essential to tumor proliferation. Moreover, the obvious reduction of P65 expressing profiles was detected in the MELK insufficient tumor confirming that NF-κB activity could be affected by MELK (Figure 6E). To confirm the effects of MELK on NF-κB methylation, lysates of tumor tissues were harvested by pull down assay with NF-κB antibody and western blot was performed. As shown in the Figure 6F, difference of Methyl K was distinctive, which indicated that MELK promoted the activation of EZH2/NF-κB complex. Taken together, the above data suggest a model that phosphorylation of EZH2 mediated by MELK promotes the GSCs self-renewal through NF-κB methylation (Figure 6G).

## Discussion

Although many studies discussed proteins and pathways driving tumor initiation and progression, little is known about specific molecular mechanisms required for GSCs self-renewal. Epigenetic reprogramming increases oncogenic potential of CSCs, which can lead to tumor growth or therapeutic resistance (10, 33). We recently demonstrated that GSCs depended on MELK/c-JUN signaling pathway for survival and to maintain an immature state (21). The current study revealed that the tumor-specific MELK activity is essential for the EZH2/NF-κB interaction via enhancing the methyltransferase activity and promotes the GSCs proliferation and maintains the stemness.

Elucidating the cooperation between MELK, EZH2, and NF-κB in GSCs is critical to understand molecular signaling mechanisms underlying GBM proliferation and progression. Given that exaggerated profiles of the MELK/EZH2/NF-κB axis are being identified in tissues with a high percentage of Ki-67^+^, such as the embryonic subventricular zone (SVZ) and in high-grade gliomas (Figure 1B and Figure S4), it seems likely that these proteins function to promote proliferation. Our findings shed light on the role of MELK/EZH2/NF-κB axis in regulating self-renewal and oncogenesis. Furthermore, because GSCs and NSCs share same signaling pathways driving their proliferation, such as Notch, Hedgehog or WNT signaling (43), it is reasonable that inducing differentiation as a therapeutic strategy could decrease the malignant potential of GBM without the severe adverse effects caused by extensive cell death. It is likely that the stem cell niche is significantly changed within neoplasms relative to the normal NSCs niche. Recent studies have discovered the vital role of tumor micro-environment, which participates in regulating the stability of a variety of molecules on GSCs self-proliferation (44). GSCs have the ability to modify the microenvironment by recruiting or generating agents to make others serve themselves with the goal of maintaining self-renewal (13, 45).

EZH2, as the epigenetic player, plays a fundamental role in supporting stem cell tumorigenicity by mediation of transcription silencing and activation of downstream transcription factors. In our study, NF-κB function was found to be promoted by EZH2 methyltransferase activity and that GSCs require NF-κB to overcome differentiation and maintain stemness by this methylation. Further, MELK plays a key role in EZH2/NF-κB activity as targeting MELK in GSCs can suppress the hyperactivation of NF-κB (Figure 6). In recent study utilizing various MELK knockouts across several cancer types, authors demonstrated that MELK deficiency didn't alter tumor growth (41). Inconsistent with our data, authors suggested that MELK might be upregulated in cancer cells and its function in cell cycle progression might be redundant with other kinases. It is worth noting that this finding doesn't exclude the possibility that MELK can also function in cancer stemness with its cell cycle dependent expression pattern. GSCs proliferate actively with the upregulated thousands of genes involved in cell cycle progression, which may explain why MELK is commonly overexpressed in CSCs.

Herein, we demonstrate that NF-κB functions downstream of the MELK/EZH2 complex, which opens another exciting pathway to better understand the mechanism of tumorigenesis, beyond the well-established Rel/NF-κB interaction (38, 42). Activation of NF-κB involves a series of sequential events including DNA binding, dimerization, nuclear importation and tyrosine phosphorylation (46, 47). Deeper understanding of molecular mechanisms associated with this pathway will enable development of targeted inhibitors and provide rationale for establishment of the most effective diagnostic and therapeutic applications. As an example, we can evaluate the inhibitory effects of EZH2 via examining the NF-κB activity. In addition to assessing the role of MELK/EZH2/NF-κB axis in different histopathological gliomas, additional studies will investigate their function in genetic subgroups of glioma. Additionally, the impacts of knockdown and inhibitors on tumor burden in our work demonstrate a role of targeted therapy for glioma, which is in agreement with the previous reports aimed to alter activity of MELK/EZH2/NF-κB axis (48).

## Conclusion

In this study, we report a critical function of MELK/EZH2/NF-κB axis in maintaining GSC stemness, as evidenced by the mechanism demonstrating that MELK-mediated EZH2 phosphorylation regulates proliferation and differentiation of GSCs through NF-κB methylation. The expression profiles of MELK, EZH2 and NF-κB represent another prognostic factor for glioma patients, further supporting the clinical application in precise diagnosis and treatment. Potentially, this strategy may apply in other cancers if the pathway functions in a similar way within other malignancy. In conclusion, the MELK/EZH2/NF-κB axis represents the exciting new prognostic and potential therapeutic target in GBM.

## Ethics Statement

This study was carried out in accordance with the recommendations of the ethics committee of Sanbo Brain Hospital Capital Medical University. All subjects gave written informed consent in accordance with the Declaration of Helsinki.

## Author Contributions

HL and CG conceived and designed the project. HL, YSu, and HY performed experimental procedures. JZ and XQ provided pathological analysis of tissues. HL, ZW, and JO contributed to the analysis and interpretation of data. MZ, JZ, and CG worked as the neurosurgeons to collect all the current cases and analyze the clinical data. HL and CG completed the manuscript, figures, and tables, as well as YSo, CY, and RG made valuable comments and edited the manuscript.

### Conflict of Interest Statement

The authors declare that the research was conducted in the absence of any commercial or financial relationships that could be construed as a potential conflict of interest.

## Supplementary Material

Figure S1 **(A)** Pearson analysis showing the correlation of EZH2 with MELK or NF-κB expression (Pearson *r* = 0.89 and 0.76, respectively). **(B)** The postsurgical OS and PFS curves evaluated by Kaplan-Meier method among the whole cases showing poor survival was associated with high expression of MELK, EZH2, or NF-κB (low, the IHC score of MELK, EZH2, or NF-κB was <0.7, 1.0, and 1.0, respectively; high, the IHC score was more than 0.8, 1.1, and 1.1, respectively). **(C)** The OS and PFS curves evaluated by Kaplan-Meier analysis based on histological grades indicating that patients with lower grades had longer survival.

Figure S2 Scheme presenting the procedure of isolation of GSCs and the immunostaining showing the expression of CD133 and Nestin in sorted GSCs.

Figure S3 Western blot analysis showing the higher expression of MELK, EZH2, NF-κB, and Nestin in GSCs (U87 and 8,111) compared with the differentiated progeny.

Figure S4 **(A)** Immunostaining of MELK and EZH2 in sagittal sections from mouse brains (E18.5, P8.5, and P28.5) showing gradually decreased expression of MELK/EZH2 at SVZ and hippocampus (100× and 400×). **(B)** Immunostaining showing the expression of NF-κB at SVZ of E18.5 mouse and merged with Sox2 expression, but not at the mature SVZ (P30.5, 100×, and 400×). **(C)** IHC staining showing high Ki-67 index at the E18.5 SVZ and P8.5 hippocampus (100× and 400×).

Figure S5 qPCR analysis showing no significant difference in NF-κB mRNA expression between EZH2 inhibition and control group.

Figure S6 **(A)** Subcutaneous xenografts volumes diagram showing tumor growth was inhibited after treating with EZH2/NF-κB inhibitors. **(B)** Immunostaining showing the reductive Ki-67 index across the xenografts after using the EZH2 or NF-κB inhibitors (100×). ^**^*p* < 0.01, ^***^*p* < 0.001.

Figure S7 **(A)** Immunostaining of Nestin showing the decreased GSCs spheres formation after MELK knockdown or OTSSP167 treatment (200×). **(B)** The tumor growth rate curves showing the decreased growth of xenografts derived from MELK deficiency GSCs. **(C)** H.E. staining showing the Grade 2 morphology in MELK knockdown xenografts and immunostaining showing the expression of Ki-67, GFAP and Nestin in the xenografts arising from MELK deficient GSCs or addition with IL-1 (100×). ^*^*p* < 0.05, ^**^*p* < 0.01.

Figure S8 **(A)** Tumor volumes diagram showing the decreased tumor growth after treating with OTSSP167 or ACHP. **(B)** Immunostaining showing the declined Ki-67 labeling in subcutaneous tumors treated with OTSSP167 or ACHP (100×). ^*^*p* < 0.05, ^**^*p* < 0.01, ^***^*p* < 0.001.

Table S1 Clinicopathological characters of patients with glioma.

Table S2 Log-rank test and Logistic estimates for survival of glioma patients.

Table S3 Univariate and multivariate analysis using Cox proportional hazards model.

Data Sheet 1 Supplementary materials and methods.
