# Supplementary figures and images for: EZH2 Phosphorylation Promotes Self-Renewal of Glioma Stem-Like Cells Through NF-κB Methylation
Source: Front Oncol. 2019 Jul 16;9:641. doi: 10.3389/fonc.2019.00641 (PMC6652807; doi:10.3389/fonc.2019.00641)

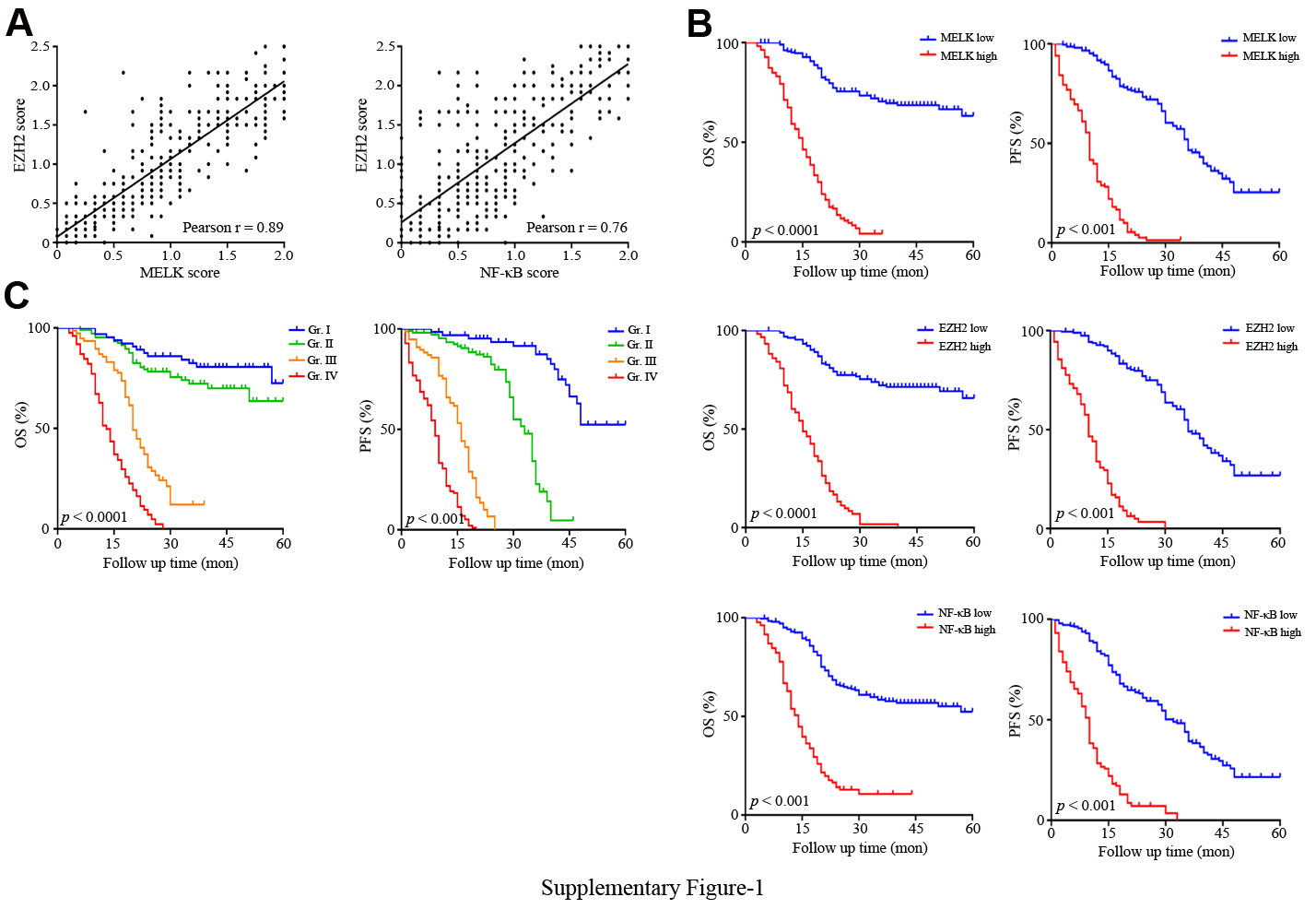

Supplement: Figure S1 — (A) Pearson analysis showing the correlation of EZH2 with MELK or NF-κB expression (Pearson r = 0.89 and 0.76, respectively). (B) The postsurgical OS and PFS curves evaluated by Kaplan-Meier method among the whole cases showing poor survival was associated with high expression of MELK, EZH2, or NF-κB (low, the IHC score of MELK, EZH2, or NF-κB was <0.7, 1.0, and 1.0, respectively; high, the IHC score was more than 0.8, 1.1, and 1.1, respectively). (C) The OS and PFS curves evaluated by Kaplan-Meier analysis based on histological grades indicating that patients with lower grades had longer survival. [file Image_1.TIF]

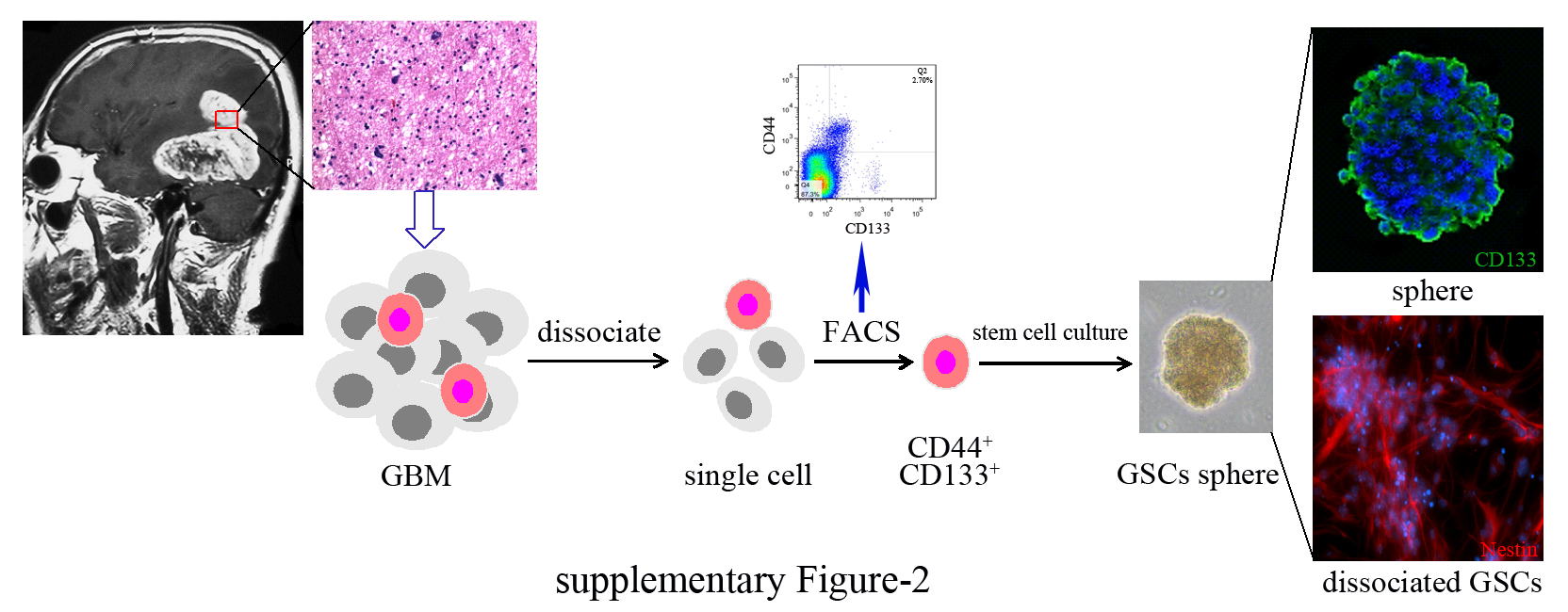

Supplement: Figure S2 — Scheme presenting the procedure of isolation of GSCs and the immunostaining showing the expression of CD133 and Nestin in sorted GSCs. [file Image_2.TIF]

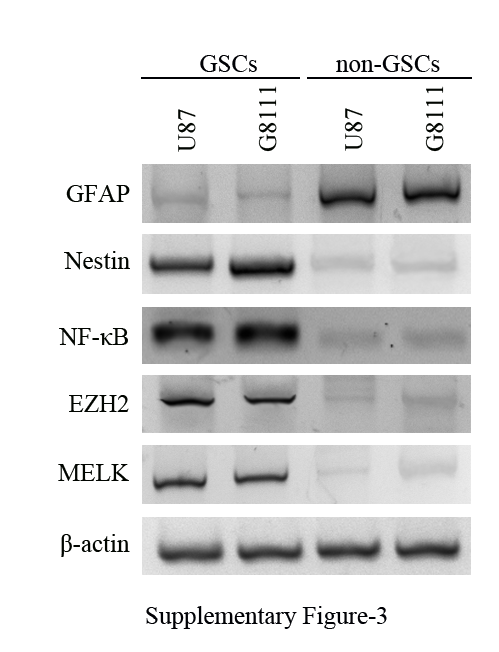

Supplement: Figure S3 — Western blot analysis showing the higher expression of MELK, EZH2, NF-κB, and Nestin in GSCs (U87 and 8,111) compared with the differentiated progeny. [file Image_3.TIF]

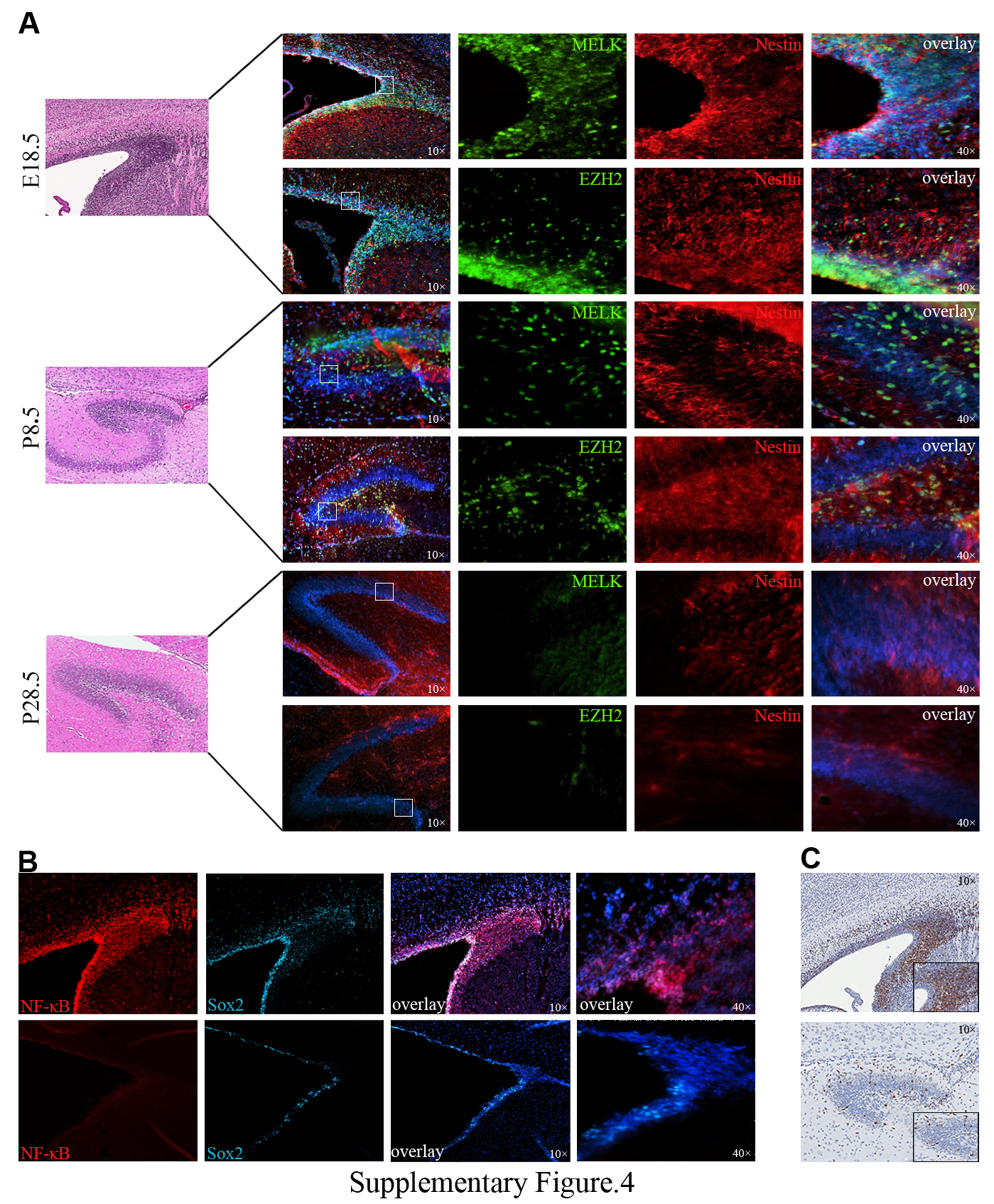

Supplement: Figure S4 — (A) Immunostaining of MELK and EZH2 in sagittal sections from mouse brains (E18.5, P8.5, and P28.5) showing gradually decreased expression of MELK/EZH2 at SVZ and hippocampus (100× and 400×). (B) Immunostaining showing the expression of NF-κB at SVZ of E18.5 mouse and merged with Sox2 expression, but not at the mature SVZ (P30.5, 100×, and 400×). (C) IHC staining showing high Ki-67 index at the E18.5 SVZ and P8.5 hippocampus (100× and 400×). [file Image_4.jpg]

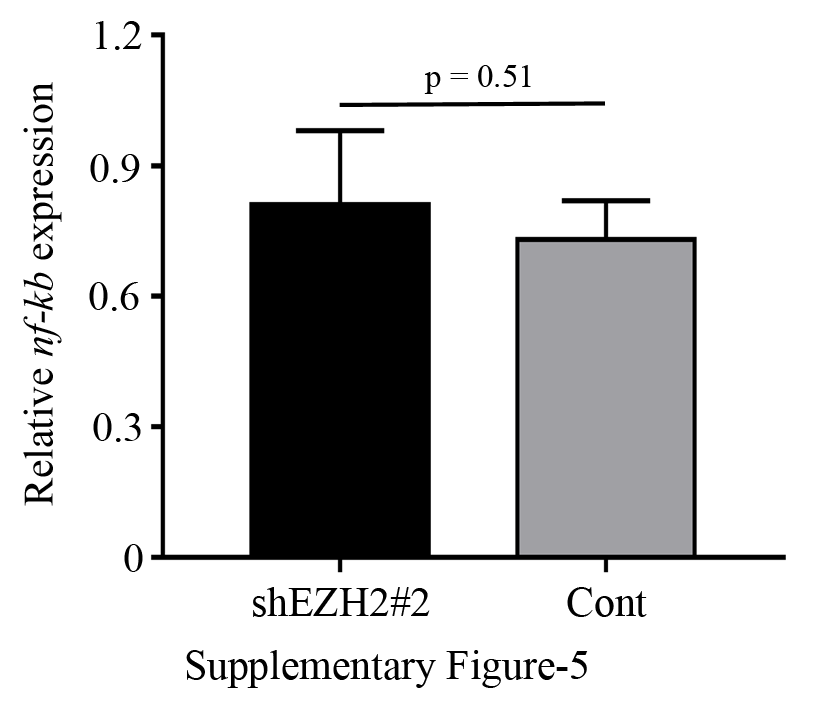

Supplement: Figure S5 — qPCR analysis showing no significant difference in NF-κB mRNA expression between EZH2 inhibition and control group. [file Image_5.TIF]

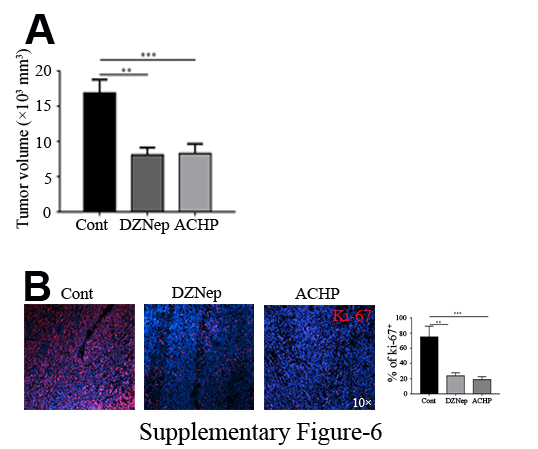

Supplement: Figure S6 — (A) Subcutaneous xenografts volumes diagram showing tumor growth was inhibited after treating with EZH2/NF-κB inhibitors. (B) Immunostaining showing the reductive Ki-67 index across the xenografts after using the EZH2 or NF-κB inhibitors (100×). **p < 0.01, ***p < 0.001. [file Image_6.TIF]

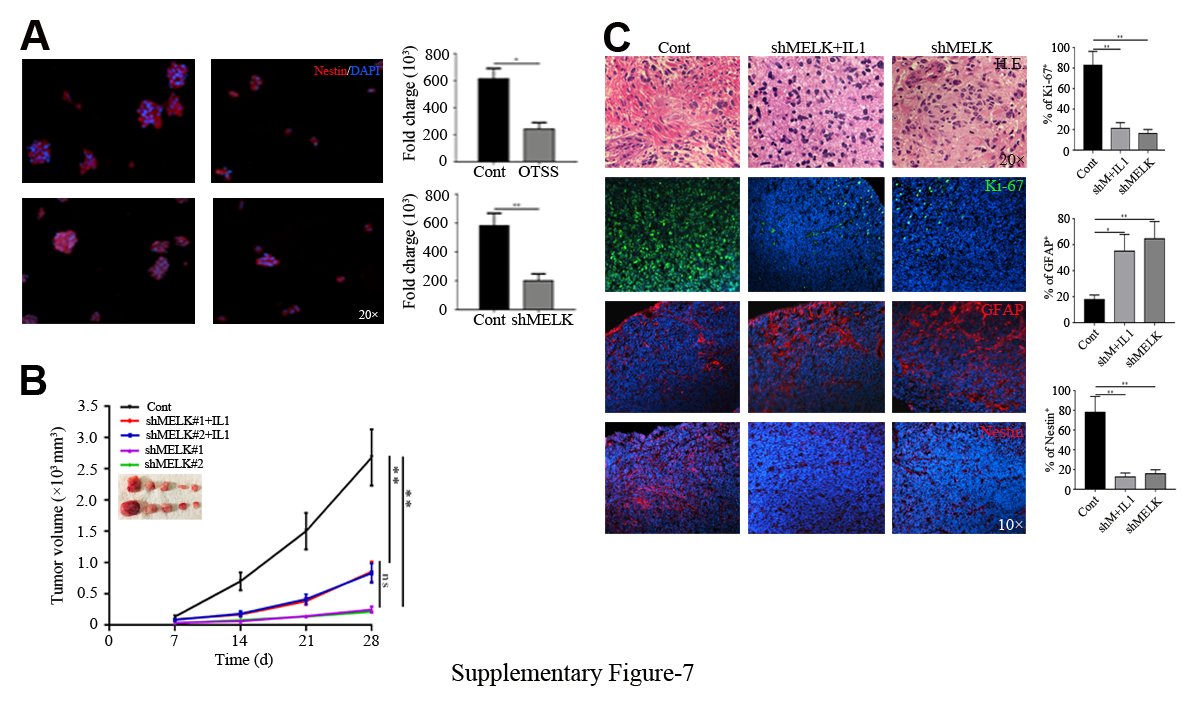

Supplement: Figure S7 — (A) Immunostaining of Nestin showing the decreased GSCs spheres formation after MELK knockdown or OTSSP167 treatment (200×). (B) The tumor growth rate curves showing the decreased growth of xenografts derived from MELK deficiency GSCs. (C) H.E. staining showing the Grade 2 morphology in MELK knockdown xenografts and immunostaining showing the expression of Ki-67, GFAP and Nestin in the xenografts arising from MELK deficient GSCs or addition with IL-1 (100×). *p < 0.05, **p < 0.01. [file Image_7.TIF]

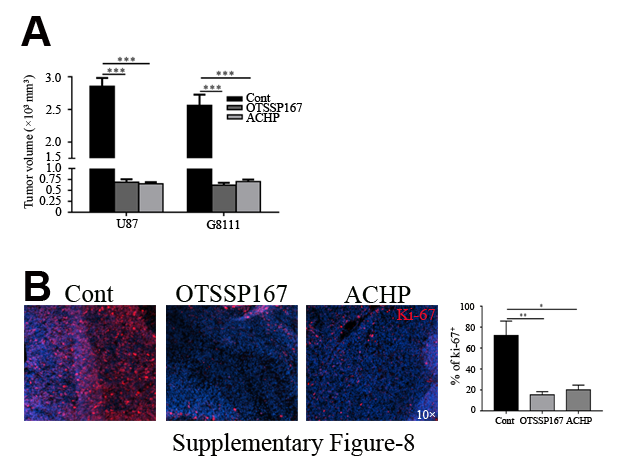

Supplement: Figure S8 — (A) Tumor volumes diagram showing the decreased tumor growth after treating with OTSSP167 or ACHP. (B) Immunostaining showing the declined Ki-67 labeling in subcutaneous tumors treated with OTSSP167 or ACHP (100×). *p < 0.05, **p < 0.01, ***p < 0.001. [file Image_8.TIF]
