# Supplementary material for: EZH2 Phosphorylation Promotes Self-Renewal of Glioma Stem-Like Cells Through NF-κB Methylation
Source: Front Oncol. 2019 Jul 16;9:641. doi: 10.3389/fonc.2019.00641 (PMC6652807; doi:10.3389/fonc.2019.00641)
Supplement: Table S1 — Clinicopathological characters of patients with glioma. [file Table_1.DOC]

**Table S1. Clinicopathological characters of patients with glioma**

| **parameters** | **No. (%)** | **MELK** | | | ***p***  **value** | **EZH2** | | | ***p***  **value** | **NF-κB** | | | ***p***  **value** |
| --- | --- | --- | --- | --- | --- | --- | --- | --- | --- | --- | --- | --- | --- |
|  |  | < 1.0 | 1.0 - 1.5 | > 1.5 |  | < 1.0 | 1.0 - 1.5 | > 1.5 |  | < 1.0 | 1.0 - 1.5 | > 1.5 |  |
| **Gender** |  |  |  |  | 0.23 |  |  |  | 0.52 |  |  |  | 0.15 |
| Male | 206 (54.93) | 87 (23.20) | 73 (19.47) | 46 (12.27) |  | 89 (23.73) | 62 (16.53) | 55 (14.67) |  | 108 (28.80) | 77 (20.53) | 21 (5.60) |  |
| Female | 169 (45.07) | 62 (16.53) | 69 (18.40) | 38 (10.13) |  | 71 (18.93) | 44 (11.73) | 54 (14.40) |  | 91 (24.27) | 59 (15.73) | 19 (5.07) |  |
| **Age (y)** |  |  |  |  | 0.08 |  |  |  | 0.12 |  |  |  | 0.33 |
| < 35.0 | 165 (44.00) | 78 (20.80) | 51 (13.60) | 36 (9.60) |  | 85 (22.67) | 37 (9.87) | 43 (11.47) |  | 98 (26.13) | 51 (13.60) | 16 (4.27) |  |
| ≥ 35.0 | 210 (56.00) | 71 (18.93) | 91 (24.27) | 48 (12.80) |  | 75 (20.00) | 69 (18.40) | 66 (17.60) |  | 101 (26.93) | 85 (22.67) | 24 (6.40) |  |
| **Prognosis** |  |  |  |  | 0.03 |  |  |  | 0.01 |  |  |  | < 0.01 |
| Alive | 193 (51.47) | 122 (32.53) | 59 (15.73) | 12 (3.20) |  | 129 (34.40) | 42 (11.20) | 22 (5.87) |  | 141 (37.60) | 48 (12.80) | 4 (1.07) |  |
| Dead | 182 (48.53) | 27 (7.20) | 83 (22.13) | 72 (19.20) |  | 31 (8.27) | 64 (17.07) | 87 (23.20) |  | 58 (15.47) | 88 (23.47) | 36 (9.60) |  |
| **Pathological grade** |  |  |  |  | < 0.001 |  |  |  | < 0.001 |  |  |  | 0.02 |
| I - II | 173 (46.13) | 139 (37.07) | 34 (9.07) | 0 |  | 148 (39.47) | 25 (6.67) | 0 |  | 143 (38.13) | 30 (8.00) | 0 |  |
| III - IV | 202 (53.87) | 10 (2.67) | 108 (28.80) | 84 (22.40) |  | 12 (3.20) | 81 (21.60) | 109 (29.07) |  | 56 (14.93) | 106 (28.27) | 40 (10.67) |  |
| **Location** |  |  |  |  | < 0.01 |  |  |  | 0.04 |  |  |  | < 0.001 |
| Frontal lobe | 137 (36.53) | 36 (9.60) | 72 (19.20) | 29 (7.73) |  | 39 (10.40) | 48 (12.80) | 50 (13.33) |  | 57 (15.20) | 69 (18.40) | 11 (2.93) |  |
| Temporal lobe | 78 (20.80) | 34 (9.07) | 26 (6.93) | 18 (4.80) |  | 35 (9.33) | 20 (5.33) | 23 (6.13) |  | 48 (12.80) | 21 (5.60) | 9 (2.40) |  |
| Parietal lobe | 9 (2.40) | 5 (1.33) | 4 (1.07) | 0 |  | 7 (1.87) | 2 (0.53) | 0 |  | 5 (1.33) | 4 (1.07) | 0 |  |
| Occipital lobe | 23 (6.13) | 5 (1.33) | 5 (1.33) | 13 (3.47) |  | 4 (1.07) | 4 (1.07) | 15 (4.00) |  | 6 (1.60) | 10 (2.67) | 7 (1.87) |  |
| Basal ganglia | 26 (6.93) | 14 (3.73) | 7 (1.87) | 5 (1.33) |  | 12 (3.20) | 10 (2.67) | 4 (1.07) |  | 17 (4.53) | 7 (1.87) | 2 (0.53) |  |
| sellar region | 24 (6.40) | 15 (4.00) | 7 (1.87) | 2 (0.53) |  | 17 (4.53) | 5 (1.33) | 2 (0.53) |  | 15 (4.00) | 7 (1.87) | 2 (0.53) |  |
| Cerebellum | 22 (5.87) | 15 (4.00) | 6 (1.60) | 1 (0.27) |  | 17 (4.53) | 4 (1.07) | 1 (0.27) |  | 18 (4.80) | 3 (0.80) | 1 (0.27) |  |
| Brainstem | 39 (10.40) | 17 (4.53) | 10 (2.67) | 12 (3.20) |  | 20 (5.33) | 9 (2.40) | 10 (2.67) |  | 22 (5.87) | 12 (3.20) | 5 (1.33) |  |
| **PTE** |  |  |  |  | 0.06 |  |  |  | 0.11 |  |  |  | 0.34 |
| Gr. I | 146 (38.93) | 128 (34.13) | 18 (4.80) | 0 |  | 127 (33.87) | 17 (4.53) | 2 (0.53) |  | 128 (34.13) | 18 (4.80) | 0 |  |
| Gr. II | 54 (14.40) | 14 (3.73) | 34 (9.07) | 6 (1.60) |  | 20 (5.33) | 23 (6.13) | 11 (2.93) |  | 30 (8.00) | 23 (6.13) | 1 (0.27) |  |
| Gr. III | 175 (46.67) | 7 (1.87) | 90 (24.00) | 78 (20.80) |  | 13 (3.47) | 66 (17.60) | 96 (25.60) |  | 41 (10.93) | 95 (25.33) | 39 (10.40) |  |
| **MRI Enhancement** |  |  |  |  | 0.03 |  |  |  | < 0.001 |  |  |  | 0.02 |
| None | 13 (3.47) | 13 (3.47) | 0 | 0 |  | 13 (3.47) | 0 | 0 |  | 13 (3.47) | 0 | 0 |  |
| Homo- | 59 (15.73) | 40 (10.67) | 16 (4.27) | 3 (0.80) |  | 41 (10.93) | 13 (3.47) | 5 (1.33) |  | 49 (13.07) | 10 (2.67) | 0 |  |
| Heter- | 303 (80.80) | 96 (25.60) | 126 (33.60) | 81 (21.60) |  | 106 (28.27) | 93 (24.80) | 104 (27.73) |  | 137 (36.53) | 126 (33.60) | 40 (10.67) |  |
| **Ki-67** |  |  |  |  | < 0.0001 |  |  |  | < 0.001 |  |  |  | < 0.001 |
| < 10.0% | 133 (35.47) | 121 (32.27) | 11 (2.93) | 1 (0.27) |  | 123 (32.80) | 7 (1.87) | 3 (0.80) |  | 122 (32.53) | 11 (2.93) | 0 |  |
| 10.0 - 25.0% | 163 (43.47) | 28 (7.47) | 121 (32.27) | 14 (3.73) |  | 36 (9.60) | 93 (24.80) | 34 (9.07) |  | 77 (20.53) | 85 (22.67) | 1 (0.27) |  |
| > 25.0% | 79 (21.07) | 0 | 10 (2.67) | 69 (18.40) |  | 1 (0.27) | 6 (1.60) | 72 (19.20) |  | 0 | 40 (10.67) | 39 (10.40) |  |

Data was presented as number (percentage). PTE, peritumoral edema.
