# Supplementary material for: EZH2 Phosphorylation Promotes Self-Renewal of Glioma Stem-Like Cells Through NF-κB Methylation
Source: Front Oncol. 2019 Jul 16;9:641. doi: 10.3389/fonc.2019.00641 (PMC6652807; doi:10.3389/fonc.2019.00641)
Supplement: Table S2 — Log-rank test and Logistic estimates for survival of glioma patients. [file Table_2.DOC]

**Table S2. Kaplan-Meier and Logistic estimates for survival of glioma patients**

| **Variable** | **No. (%)** | **Survival rate** | **Log-rank** | **OR** |
| --- | --- | --- | --- | --- |
| **Gender** |  |  | 0.32 | 0.45 |
| Male | 206 (54.93) | 109 (52.91) |  |  |
| Female | 169 (45.07) | 84 (49.70) |  |  |
| **Age (y)** |  |  | 0.07 | 1.02 |
| < 35.0 | 165 (44.00) | 100 (60.61) |  |  |
| ≥ 35.0 | 210 (56.00) | 93 (44.29) |  |  |
| **Pathological grade** |  |  | 0.002 | 2.11 |
| I - II | 173 (46.13) | 132 (76.30) |  |  |
| III - IV | 202 (53.87) | 61 (30.20) |  |  |
| **Location** |  |  | < 0.001 | 1.86 |
| Cerebera | 247 (65.87) | 119 (48.18) |  |  |
| Basal ganglia | 26 (6.93) | 13 (50.00) |  |  |
| sellar region | 24 (6.40) | 17 (70.83) |  |  |
| Cerebellum | 22 (5.87) | 14 (63.64) |  |  |
| Brainstem | 39 10.40) | 24 (61.54) |  |  |
| **PTE** |  |  | 0.11 | 1.13 |
| Gr. I | 146 (38.93) | 122 (83.56) |  |  |
| Gr. II | 54 (14.40) | 30 (55.56) |  |  |
| Gr. III | 175 (46.67) | 41 (23.43) |  |  |
| **MRI Enhancement** |  |  | 0.03 | 1.67 |
| None | 13 (3.47) | 12 (92.31) |  |  |
| Homo- | 59 (15.73) | 43 (72.88) |  |  |
| Heter- | 303 (80.80) | 138 (45.54) |  |  |
| **Ki-67** |  |  | < 0.0001 | 1.96 |
| < 10.0% | 133 (35.47) | 121 (90.98) |  |  |
| 10.0 - 25.0% | 163 (43.47) | 62 (38.04) |  |  |
| > 25.0% | 79 (21.07) | 10 (12.66) |  |  |
| **MELK status** |  |  | < 0.001 | 1.85 |
| < 1.0 | 149 (39.73) | 122 (81.88) |  |  |
| 1.0 - 1.5 | 142 (37.87) | 59 (41.55) |  |  |
| > 1.5 | 84 (22.40) | 12 (14.29) |  |  |
| **EZH2 status** |  |  | < 0.0001 | 2.26 |
| < 1.0 | 160 (42.67) | 129 (80.63) |  |  |
| 1.0 - 1.5 | 106 (28.27) | 42 (39.62) |  |  |
| > 1.5 | 109 (29.07) | 22 (20.18) |  |  |
| **NF-κB status** |  |  | < 0.0001 | 1.76 |
| < 1.0 | 199 (53.07) | 141 (70.85) |  |  |
| 1.0 - 1.5 | 136 (36.27) | 48 (35.29) |  |  |
| > 1.5 | 40 (10.67) | 4 (10.00) |  |  |

Data was presented as number (percentage). PTE, peritumoral edema.
