# Supplementary material for: EZH2 Phosphorylation Promotes Self-Renewal of Glioma Stem-Like Cells Through NF-κB Methylation
Source: Front Oncol. 2019 Jul 16;9:641. doi: 10.3389/fonc.2019.00641 (PMC6652807; doi:10.3389/fonc.2019.00641)
Supplement: Table S3 — Univariate and multivariate analysis using Cox proportional hazards model. [file Table_3.DOC]

**Table S3. Univariate and multivariate analysis using Cox proportional hazards model**

| **Variable** | **Univariate** | | **Multivariate** | |
| --- | --- | --- | --- | --- |
|  | **HR (95% CI)** | p value | **HR (95% CI)** | p value |
| **Gender (M/F)** | 1.231 (0.623-1.901) | 0.352 |  |  |
| **Age (≤ 35.0y/> 35.0y)** | 0.759 (0.393-1.277) | 0.08 |  |  |
| **Pathological grade (L/H)** | 1.975 (1.206-2.850) | < 0.0001 | 0.784 (0.326-1.372) | 0.014 |
| **Location** | 2.587 (1.233-4.003) | < 0.001 | 1.836 (1.025-2.990) | < 0.001 |
| **PTE (I-II/III)** | 0.525 (0.312-0.952) | 0.267 |  |  |
| **Enhancement (non/yes)** | 1.582 (0.983-2.431) | 0.002 | 2.035 (1.239-2.874) | < 0.01 |
| **Ki-67 (<9.0%/>10.0%)** | 2.459 (1.335-3.573) | < 0.0001 | 2.015 (0.952-3.563) | < 0.001 |
| **MELK profile (neg/pos)** | 2.461 (1.536-3.882) | < 0.0001 | 0.716 (0.325-1.202) | < 0.0001 |
| **EZH2 profile (neg/pos)** | 1.579 (0.659-2.650) | < 0.001 | 0.692 (0.214-1.106) | 0.011 |
| **NF-κB profile (neg/pos)** | 1.378 (1.021-1.786) | 0.003 | 1.578 (0.643-2.531) | < 0.01 |

PTE, peritumoral edema.
