# Supplementary material for: EZH2 Phosphorylation Promotes Self-Renewal of Glioma Stem-Like Cells Through NF-κB Methylation
Source: Front Oncol. 2019 Jul 16;9:641. doi: 10.3389/fonc.2019.00641 (PMC6652807; doi:10.3389/fonc.2019.00641)
Supplement: Data Sheet 1 — Supplementary materials and methods. [file Data_Sheet_1.doc]

**Supplementary materials and methods**

**Neuroradiological parameters of the whole cases**

On MRI scans, enhancement was classified as none, heterogeneous and homogeneous features according to the appearance on T1-weight image (T1WI) with gadolinium. Peritumoral edema (PTE) on axial T2WI was analyzed on the basis of Asari’s method and defined as three grades: Gr Ⅰ, PTE diameter was less than that of tumor; Gr Ⅱ, PTE diameter was more than or equal to tumor diameter but less than two times; Gr Ⅲ, PTE diameter was two times more than or equal to tumor diameter. Tumor location was identified based on the anatomic regions as listed in Table S1. Two observers at the Neuroimaging Department of our institute independently interpreted the MRI scans.

**Cell-line**

Human GBM U87 cell-line was obtained from National Infrastructure of Cell Line Resource in Peking Union Medical College (PUMC) in Jun 2013. Primary human GBM cells were established from fresh specimens in our institute, following the protocol of digestion with papain (20 U/ml) and then resuspending at 1 × 10^7^ /ml in DMEM medium with 10% FBS at 37 ℃ in a humidified 5% CO_2_ incubator.

**Main reagents**

OTSSP167 (MELK inhibitor, Millipore 532604), DZNep (EZH2 inhibitor, Sigma252790) and ACHP (NF-κB inhibitor, MCE HY-13060) were used in current study. Papain, DNase Ⅰ and B27 were purchased from Sigma company. Following reagents were purchased from Gibco company containing Neurobasal medium, DMEM medium, DMEM/F12 medium, penicillin/streptomycin, basic fibroblast growth factor (bFGF), epidermal growth factor (EGF) and L-glutamine.

**Western blot**

The lyse splitting buffer composed the Triton X-100 (0.1%), Tris-HCl (20 mM, pH = 7.4) and protease-inhibitor cocktail. The immunoblotting antibodies included anti-MELK, EZH2, NF-κB, Nestin, GFAP or β-actin antibodies and anti-SUV391H1, p-KMT6/EZH2 (Thr487), and H3K27me3 antibodies (Abcam Biotechnology) and all of first antibodies were diluted at 1︰500 expect for β-actin at 1︰3000.

**qPCR**

The primer sequences were listed as follows,

MELK: 5’-CACCGCAGCAGCAGGCAGAC-3’, 3’-GGGTTGGTGAGGCGGGTATTTC-5’;

EZH2: 5’-CCCTTCTCAGATTTCTTCCCA-3’, 3’-GAGGTGACGGAAGACTCAGG-5’;

NF-κB: 5’-GTGGGGACTACGACCTGAATG-3’, 3’-GGGGCACGATTGTCAAAGATG-5’.
